# Radiogenomic analysis of cellular tumor-stroma heterogeneity as a prognostic predictor in breast cancer

**DOI:** 10.1186/s12967-023-04748-6

**Published:** 2023-11-25

**Authors:** Ming Fan, Kailang Wang, You Zhang, Yuanyuan Ge, Zhong Lü, Lihua Li

**Affiliations:** 1 Institute of Intelligent Biomedicine, Hangzhou Dianzi University, Hangzhou, 310018 China; 2 Affiliated Dongyang Hospital of Wenzhou Medical University, Dongyang, 322100 China

**Keywords:** Breast cancer, Cell subpopulation, Prognosis, Radiogenomics

## Abstract

**Background:**

The tumor microenvironment and intercellular communication between solid tumors and the surrounding stroma play crucial roles in cancer initiation, progression, and prognosis. Radiomics provides clinically relevant information from radiological images; however, its biological implications in uncovering tumor pathophysiology driven by cellular heterogeneity between the tumor and stroma are largely unknown. We aimed to identify radiogenomic signatures of cellular tumor-stroma heterogeneity (TSH) to improve breast cancer management and prognosis analysis.

**Methods:**

This retrospective multicohort study included five datasets. Cell subpopulations were estimated using bulk gene expression data, and the relative difference in cell subpopulations between the tumor and stroma was used as a biomarker to categorize patients into good- and poor-survival groups. A radiogenomic signature-based model utilizing dynamic contrast-enhanced magnetic resonance imaging (DCE-MRI) was developed to target TSH, and its clinical significance in relation to survival outcomes was independently validated.

**Results:**

The final cohorts of 1330 women were included for cellular TSH biomarker identification (n = 112, mean age, 57.3 years ± 14.6) and validation (n = 886, mean age, 58.9 years ± 13.1), radiogenomic signature of TSH identification (n = 91, mean age, 55.5 years ± 11.4), and prognostic (n = 241) assessments. The cytotoxic lymphocyte biomarker differentiated patients into good- and poor-survival groups (*p* < 0.0001) and was independently validated (*p* = 0.014). The good survival group exhibited denser cell interconnections. The radiogenomic signature of TSH was identified and showed a positive association with overall survival (*p* = 0.038) and recurrence-free survival (*p* = 3 × 10^–4^).

**Conclusion:**

Radiogenomic signatures provide insights into prognostic factors that reflect the imbalanced tumor-stroma environment, thereby presenting breast cancer-specific biological implications and prognostic significance.

**Supplementary Information:**

The online version contains supplementary material available at 10.1186/s12967-023-04748-6.

## Introduction

Breast cancer exhibits genetic diversity both between and within tumors, leading to diverse tumor phenotypes, disease progression, and therapeutic resistance [1, 2]. The tumor microenvironment is a complex ecosystem comprising immune cells, fibroblasts, extracellular matrix, and cytokines [3], while the surrounding stromal cells [4, 5] protect tumor cells from immune activity [6]. Altered environmental heterogeneity influences tumor initiation, progression, and response to therapy [7]. Increased tumor-infiltrating lymphocytes (TILs) have been found to be associated with chemotherapy response and improved survival [8]. Environmental heterogeneity, particularly in terms of immune cell subtypes such as CD4 cells and T cells, is associated with less activation of the immune response and poorer survival in breast cancer [9, 10]. A comprehensive understanding of tumor environmental heterogeneity is crucial for accurate prognostic assessment and effective treatment management of breast cancer.

To this end, many studies have focused on estimating the composition of multiple cell subpopulations by deconvoluting bulk tissue microarray data derived from tissue mixtures [11]. Technologies such as CIBERSORT have been developed to deconvolute large-scale RNA mixtures for the identification of cellular biomarkers and therapeutic targets [12]. By estimating the abundance of immune and stromal cell populations within heterogeneous tissues, environmental heterogeneity can be characterized [13]. Estimated cell subpopulations have been correlated with therapeutic response and survival outcomes in breast cancer [14–16]. Beyond this routine genomic approach, imaging techniques offer a noninvasive means to reveal tumor heterogeneity driven by underlying pathophysiological processes [17].

Currently, radiogenomic/radiomics studies are increasingly being performed by linking imaging features to molecular/genomic status [18–21] with the ultimate goal of improving disease management for patients [22–25]. Radiogenomic signatures of TILs have been identified and are associated with survival in patients with breast cancer [26]. Predictive imaging features that reflect cell subpopulations estimated from genomic data were identified to stratify the survival of patients [27, 28]. Tumor microenvironment-associated imaging features were used to assess chemotherapy responses and/or survival outcomes [29]. Breast cancer heterogeneity was evaluated by texture analysis of dynamic-contrast enhanced magnetic resonance imaging (DCE-MRI) and T2-weighted MRI for the prediction of survival outcomes in breast cancer [30]. All these studies demonstrated that tumor microenvironmental characteristics can be captured using noninvasive images that are ubiquitously used in clinical practice.

Emerging evidence suggests that the microenvironment is regulated through ongoing cellular crosstalk [31, 32], and interactions between tumor cells and the stroma play a crucial role in inducing an altered environment associated with cancer initiation, progression [2, 33], metastasis [34], response to neoadjuvant chemotherapy (NACT), and patient prognosis [35, 36]. While existing radiogenomic studies have predominantly focused on analyzing either tumors or the surrounding stroma, the predictive value of imaging in capturing the cellular heterogeneity between the two, which serves as a surrogate for prognosis analysis, remains uncertain.

The purpose of our study was to identify radiogenomic signatures of cellular tumor-stroma heterogeneity (TSH) and to investigate the potential of imaging TSH as a biomarker for predicting prognosis in breast cancer. In contrast to conventional data-driven studies that focus on identifying clinically relevant imaging signatures, our radiogenomic analysis delves into the cellular interactions between tumor and stroma, thereby providing additional genomic-level information with biological significance.

## Materials and methods

### Study design

**Fig. 1:**
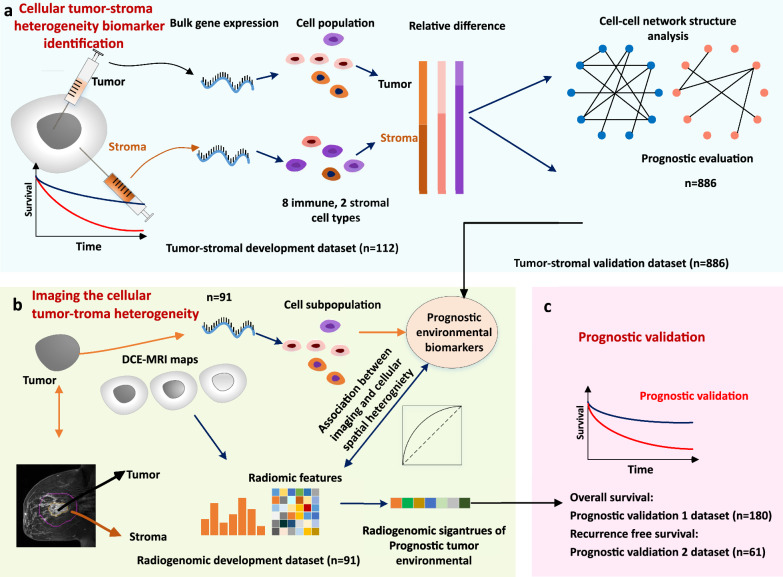
Study framework. **a** Cellular tumor-stroma heterogeneity biomarker identification. **b** Imaging the cellular tumor-stroma heterogeneity. **c** Prognostic validation. Radiogenomic analysis by associating cellular tumor-stroma biomarkers and imaging features

The framework of this three-stage study is depicted in Fig. 1. First, we estimated cell subpopulations using the microenvironment cell population counter (MCP)-counter algorithm [13]. This was accomplished by utilizing bulk gene expression data from both the tumor and surrounding stroma, forming the tumor-stroma development dataset (Fig. 1a). The cellular TSH biomarker was defined as the relative difference in cell subpopulation abundance between the tumor and stroma. Patients were separated into good- and poor-survival groups according to their cellular TSH biomarker values. Moreover, we conducted an analysis of network-level cellular connections and correlations, wherein each cell subpopulation was represented as a node, and the relationships between these nodes were defined as edges.

**Fig. 2:**
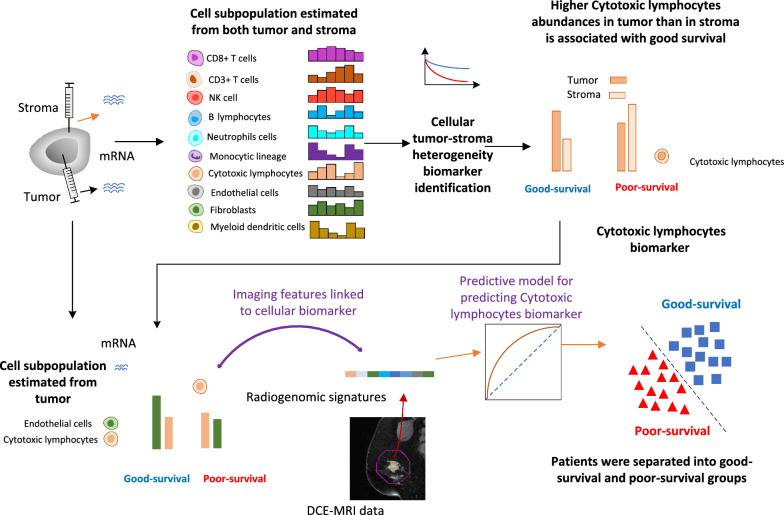
Imaging cellular tumor-stroma heterogeneity

It is important to note that to identify the radiogenomic signature of cellular TSH, it is necessary to have matched pairs of imaging and genomic data derived from both the tumor and stroma in the tumor-stroma development dataset. Unfortunately, such paired data are relatively scarce compared to data obtained solely from tumors, which presents a challenge in conducting radiogenomic analysis of TSH. Therefore, we mapped the predicted TSH using cell subpopulations derived exclusively from the tumor (Fig. 2). To validate the feasibility of our approach, we employed the tumor-stromal validation dataset comprising genomic data and follow-up data. The prognostic significance of TSH was confirmed in this dataset.

Second, the cellular biomarker-based predictive model, established using the tumor-stroma development dataset, was applied to the radiogenomic development dataset, which comprised matched imaging and genomic data (Fig. 1b). The TSH score was predicted by utilizing the estimated cell subpopulation data from the tumors. We extracted radiomic features (n = 572) from each sample and identified radiogenomic signatures by linking the imaging features derived from both the tumor and surrounding stroma with the predicted cellular TSH values. An imaging signature-based model was subsequently developed to classify patients into either good or poor survival groups.

Third, we independently validated the prognostic significance of the identified radiogenomic signatures by assessing their association with survival in prognostic validation datasets 1 and 2 (Fig. 1c).

## Datasets

**Fig. 3:**
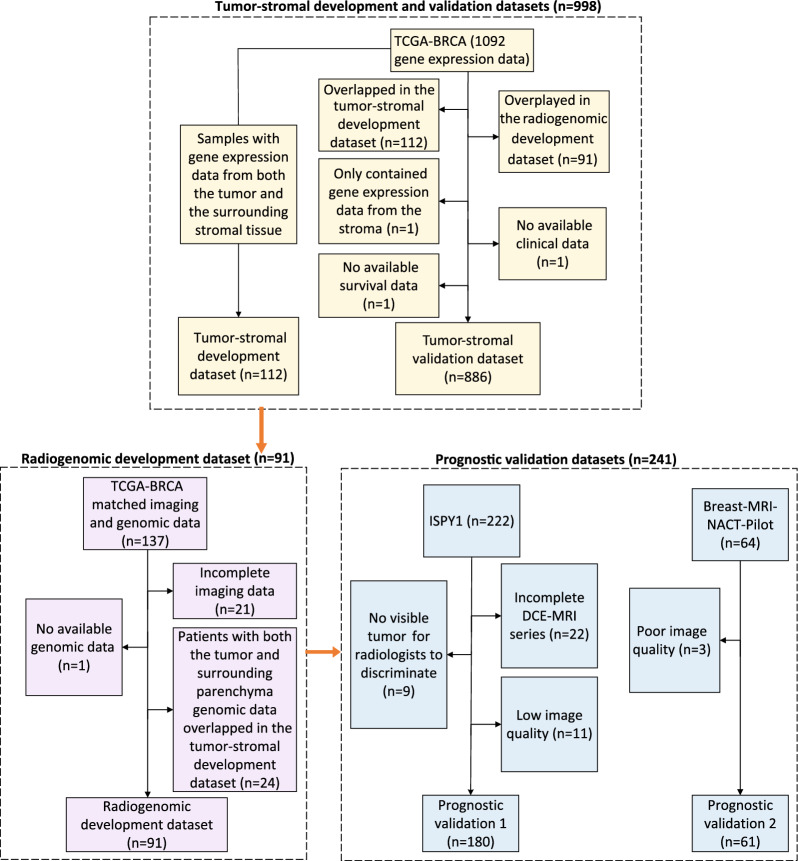
Data collection flowchart

This retrospective study included multicohort datasets of 1330 patients with genomic, imaging, and clinical data (Fig. 3). The tumor-stromal development dataset (n = 112) was collected from TCGA-BRCA [37, 38] of the Cancer Imaging Archive (TCIA) cohort. Initially, the dataset consisted of 1092 gene expression data points. We selected samples that had gene expression data from both the tumor and the surrounding parenchyma to form the tumor-stromal development dataset. This dataset was utilized to identify prognostic cell subpopulation biomarkers associated with tumor-stroma heterogeneity (TSH) and to assess cell subpopulation connectivity within the tumor or stroma.

For the tumor-stromal validation dataset (n = 886), we collected genomic data from tumors alone from TCGA-BRCA. The dataset initially included 1092 samples, while the samples overplayed in the tumor-stromal development dataset (n = 112) and the radiogenomic development dataset (n = 91) were eliminated. After excluding one patient with gene expression data solely from the parenchyma region, one patient without clinical data, and one with no available survival data, we obtained 886 samples with RNA data from tumors. This dataset served as a validation set to confirm the identified cell subpopulation biomarkers from the tumor-stroma development dataset.

The radiogenomic development dataset (n = 91) was obtained from TCGA-BRCA with matched imaging and genomic data. This dataset initially included 137 samples. After excluding one sample with unavailable genomic data and 21 samples with incomplete imaging data, we retained 115 women for analysis. We excluded 24 samples with genomic data from both the tumor and surrounding stroma, as they were utilized in the tumor-stromal development dataset. The resulting dataset (n = 91) contained tumor gene expression, matched imaging, and survival outcome data from the same patients. This dataset enabled the identification of radiogenomic signatures by linking imaging features with TSH scores.

The prognostic significance of the radiogenomic signatures was validated using two datasets with imaging data and corresponding survival information. The prognostic validation 1 (n = 61) and prognostic validation 2 (n = 180) datasets were collected from the Breast-MRI-NACT-Pilot [39] and ISPY1 [40] datasets, respectively. The prognostic validation 1 dataset initially included 64 patients. After excluding three patients owing to poor image quality, we included 61 samples for analysis. The prognostic validation 2 dataset, initially consisting of 222 women, underwent additional exclusions: 22 patients were excluded due to incomplete DCE-MRI series, nine patients were excluded because the tumors were not visually distinguishable by radiologists, and 11 patients were excluded due to poor image quality. The remaining dataset included 180 patients.

### Imaging protocols

The radiogenomic development dataset contained DCE-MRI with a T1-weighted, 3D spoiled gradient echo sequence. The imaging protocol details can be found elsewhere [20]. The DCE-MRI comprised a precontrast image series (S_0_) and three to six postcontrast image series, with a temporal resolution of approximately 110 s. The in-plane resolution ranged from 0.53–0.86 mm, the matrix was 256 × 192, the slice thickness ranged from 2.0–3.0 mm, and the flip angle was 10$^\circ$.

For the prognostic validation 1 dataset, DCE-MRI was conducted using a 1.5T fat-suppressed MRI scanner (GE Healthcare, Milwaukee, Wisconsin, USA), with patients positioned in the prone position. Further information regarding the imaging protocols can be found elsewhere [41]. The imaging parameters were as follows: repetition time (TR) = 8 ms; echo time (TE) = 8 ms; flip angle = 20°; in-plane resolution ranging from 0.7–0.9 mm; and slice thickness ranging from 2–2.4 mm. After the administration of a bolus of 0.1 mmol/kg gadobutrol, the first and second postcontrast image series were acquired at 2.5 and 7.5 min, respectively.

For the prognostic validation 2 dataset, DCE-MRI was performed using a 1.5T scanner with fat-suppressed T1-weighted imaging. The imaging parameters were set as follows: matrix = 256 × 192; field of view = 16–18 cm, TR ≤ 20 ms; TE = 4.5 ms, flip angle = 45°, slice thickness ≤ 3 mm, and in-plane resolution ≤ 1 mm. The postcontrast image series was acquired at 2.5 and 7.5 min after the administration of the contrast agent.

### Image preprocessing and feature analysis

Owing to the varied imaging protocols of the different manufacturers used in the study, each image was resized to a resolution of 0.8 × 0.8 mm and a thickness of 2 mm to ensure consistent image resolution. Subsequently, image normalization was performed by mapping each image to a standardized grayscale and dividing the pixel values by the mean value of the parenchymal region. Breast areas were obtained by excluding skin and chest walls. The stroma area was manually annotated and defined as a tissue band with a width of 20 mm (25 × 0.8 mm = 20 mm) extending from the tumor boundary [42]. The segmentation of breast tumors was performed using a spatial fuzzy c-means clustering method with the tumor’s center location as the initial “seed” being manually annotated by two experienced radiologists in consensus, each possessing 10 years of expertise [43]. To ensure the accuracy of the breast tumor segmentation results, manual correction was performed by the radiologists, which accounted for less than 5% of the segmentations.

Radiomic features (n = 107) were calculated using the publicly available Pyradiomics software package [44]. These features comprised statistical (n = 18), morphological (n = 14) and textural features (n = 75). The texture features included a gray-level cooccurrence matrix (GLCM) (n = 24), gray-level run length matrix (GLRLM, n = 16), gray-level zone length matrix (GLSZM, n = 16), gray-level difference matrix (GLDM, n = 14), and neighbourhood gray-tone difference matrix (NGTDM, n = 5). A detailed list of these features is presented in Additional file 1: Table S1.

Imaging features were calculated from the tumor and surrounding stromal tissues at different imaging phases and maps, including precontrast (S_0_), the subtraction image between the intermediate postcontrast image and S_0_ (termed S_I_), and subtraction images between the last postcontrast image and S_0_ (termed S_L_). Morphological features, on the other hand, were only calculated on a single sequence from the tumors since there were no discernible shape characteristics present in the stromal regions. In total, 572 radiogenomic imaging features were extracted from each sample.

### Cell subpopulation estimation and cell network analysis

The microenvironment cell population counter (MCP-counter) R package (http://github.com/ebecht/MCPcounter) was used to generate the absolute abundance scores of the immune and stromal cell types. The MCP-counter algorithm enables the estimation of eight immune cell types, namely, CD3+ T cells, CD8+ T cells, cytotoxic lymphocytes, NK cells, B lymphocytes, monocytic lineage, myeloid dendritic cells, and neutrophils, as well as two stromal cells, endothelial cells and fibroblasts, for comprehensive characterization of the tumor microenvironment [13]. This method was performed by comparing the gene expression profiles with predefined reference gene sets.

To ensure robust analysis, we excluded genes that displayed zero expression or were missing data in 80% of the samples. The estimation of each cell subpopulation was performed using log2-transformed gene expression data obtained from tumor and/or surrounding stromal samples.

In addition to investigating tumor-intrinsic or stromal factors, we also explored the influence of cellular biomarkers reflecting TSH, which may induce significant phenotypic alterations and influence the progression of breast cancer. The cell subpopulation biomarker associated with TSH was defined as the ratio of cell subpopulation abundance between the tumor and stromal tissue, calculated using the following formula:

$$ \frac{{A_{Tumor} - A_{Stroma} }}{{A_{Stroma} }}, $$

where ${A}_{Tumor}$ and ${A}_{Stroma}$ denote the abundance of the cell subpopulations.

In the analysis of cellular networks, a cell subpopulation was designated as a node, while the establishment of network edges was based on node similarities under a predetermined threshold (Pearson correlation coefficient > 0.5). The evaluation of network topology in the good- and poor-survival groups involved the computation of various topological parameters, including network diameter, edge number, node number, network centralization, network heterogeneity, network density, clustering coefficient, and characteristic path length. These topological parameters were calculated using widely accessible Cytoscape software (https://cytoscape.org/) to ensure accurate measurements and consistent analysis methodologies.

### Statistical analysis

Radiogenomic signatures were established by associating imaging data with predicted TSH scores using a random forest-based classifier. To mitigate multicollinearity effects, feature pairs with high similarity (r > 0.8) were removed from the predictive model. Genetic algorithm-based feature selection was employed to generate an optimal subset of features for classification. A tenfold cross-validation procedure was used to optimize feature selection and model building, reducing the risk of underfitting or overfitting.

Survival analysis was performed using the Kaplan–Meier method, obtaining hazard ratios (HRs) with 95% confidence intervals. The log-rank test was utilized to evaluate differences in survival rates between stratified groups and determine the significance of the survival curves. A multivariate Cox regression model was employed to assess the independent association of the cell subpopulations or radiogenomic signatures with overall survival (OS) or recurrence-free survival (RFS) while adjusting for the available clinical variables, including age, estrogen receptor (ER), progesterone receptor (PR), human epidermal growth factor receptor 2 (HER2), and tumor size. Patients were censored at 10 years in the absence of events. A likelihood ratio test was performed to determine whether the inclusion of radiogenomic signatures as explanatory variables significantly improved model fit compared to models based solely on clinical variables.

The Benjamin–Hochberg method was used to control for false discovery. The Wilcoxon signed-rank test was used to assess the differences in cell subpopulation abundance between the tumor and stroma. All statistical analyses were performed using MATLAB (R2018, MathWorks) and R (version 4.0; R Foundation for Statistical Computing).

## Results

### Study cohort

Five datasets comprising genomic, imaging, and clinical data were utilized for the identification and validation of TSH biomarkers, development of radiogenomic signatures, and prognostic assessments. Detailed descriptions of patient characteristics according to age, menopausal status, race, ER status, PR status, HER2 status, family history, tumor size, histological status, recurrence/survival status, and follow-up status (alive, dead, or lost to follow-up) are listed in Table 1.

**Table 1:** Patient characteristics

Parameter	Tumor-stromal development (n = 112)	Tumor-stromal validation (n = 886)	Radiogenomic development (n = 91)	Prognostic validation 1 (n = 180)	Prognostic validation 2 (n = 61)
Age					
Median	56.5 (30–90)	59 (26–90)	56 (29–82)	42.9 (26.7–68.8)	48 (29.7–72.4)
Mean ± SD	57.3 ± 14.6	58.9 ± 13.1	55.5 ± 11.4	47.7 ± 8.8	48.1 ± 9.8
ER					
Positive	78 (70)	650 (73)	75 (82)	101 (56)	28 (46)
Negative	21 (19)	200 (23)	16 (18)	77 (43)	20 (33)
N/A	13 (11)	36 (4)	0	2 (1)	13 (21)
PR					
Positive	68 (61)	560 (63)	66 (73)	84 (47)	22 (36)
Negative	31 (28)	287 (32)	25 (27)	94 (52)	26 (43)
N/A	13 (11)	39 (5)	0	2 (1)	13 (21)
HER2					
Positive	23 (21)	128 (14)	13 (14)	52 (29)	14 (23)
Negative	56 (50)	459 (52)	44 (48)	124 (69)	31 (51)
N/A	33 (29)	299 (34)	34 (38)	4 (2)	16 (26)
Follow-up (years)					
Median	3.45 (0.0–10.8)	1.98 (0.0–23.6)	2.99 (0.5–9.4)	3.90(0.5–6.8)	5.39 (0.3–9.8)
Mean ± SD	3.92 ± 2.37	3.23 ± 3.39	3.66 ± 2.11	3.84 ± 1.45	4.77 ± 2.75
Recurrence					
Event	NA	NA	NA	49(27)	23(38)
No-event	NA	NA	NA	131(73)	38(62)
Death					
Event	43 (38)	97 (11)	2 (2)	33 (18)	NA
No-event	69 (62)	789 (89)	89 (98)	143 (80)	NA
Unknown	0	0	0	4 (2)	NA

### Cellular tumor-stroma heterogeneity biomarker identification

The estimated cell subpopulations from tumors, including T cells, CD8 T cells, cytotoxic lymphocytes, B lineage cells, and myeloid dendritic cells, exhibited a significant positive association with good OS (corrected p < 0.05) (Additional file 1: Table S2). Conversely, the neutrophil cell subpopulation from the surrounding nontumoral tissue was negatively correlated with poor OS (corrected p < 0.05) (Additional file 1: Table S3), consistent with previous research [45].

Univariate analysis of the cellular TSH biomarker revealed significant positive associations between T cells, cytotoxic lymphocytes, B-lineage cells, NK cells, myeloid dendritic cells, and good OS (corrected p < 0.05). A higher abundance of these cell subpopulations within the tumor, compared to the stroma, was indicative of favorable survival outcomes (Table 2). Notably, the cytotoxic lymphocyte biomarker retained its independent association with breast cancer patient survival in the multivariable survival analysis (HR = 0.13, p = 0.017) (Additional file 1: Table S4).

**Table 2:** Prognostic assessment of the relative tumor-stroma cell subpopulation

Feature	Beta	HR (95% CI)	Wald	p value	Corrected p
T cells	− 0.96	0.38 (0.207–0.703)	9.54	0.002	0.01
CD8 T cells	-0.06	0.94 (0.823–1.084)	0.66	0.415	0.415
Cytotoxic lymphocytes	− 1.78	0.17 (0.066–0.426)	14.07	0.00018	0.002
B lineage	− 0.57	0.57 (0.357–0.895)	5.93	0.015	0.037
NK cells	− 0.86	0.42 (0.206–0.875)	5.39	0.02	0.04
Monocytic lineage	− 0.75	0.47 (0.160–1.393)	1.85	0.174	0.222
Myeloid dendritic cells	− 0.90	0.41 (0.218–0.755)	8.09	0.0045	0.015
Neutrophils	− 2.26	0.10 (0.012–0.898)	4.23	0.04	0.066
Endothelial cells	− 0.82	0.44 (0.107–1.810)	1.3	0.255	0.283
Fibroblasts	− 1.07	0.34 (0.072–1.625)	1.82	0.177	0.222

**Fig. 4:**
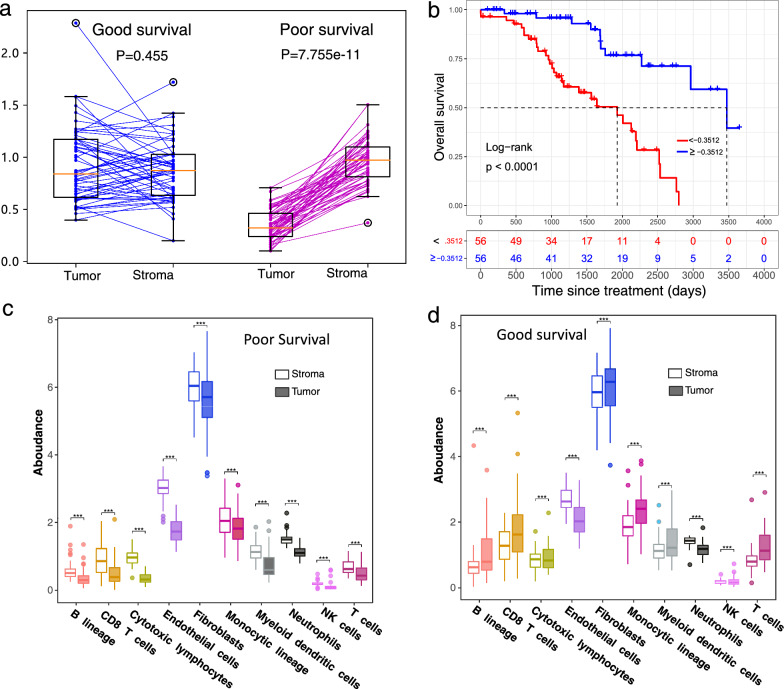
Distribution of cell subpopulations within and surrounding tumors in the good-survival and poor-survival groups. **a** The distributions of the abundance of cytotoxic lymphocyte cell subpopulations from tumor and stromal tissues in the good- and poor-survival groups. **b** Survival curves for the abundance of cytotoxic lymphocyte cell subpopulations. **c** Boxplots of cell subpopulation abundances in tumors and stromal regions in the poor-survival group. **d** Boxplots of cell subpopulation abundances in tumors and stromal regions in the good-survival group. In the boxplot, the centerline indicates the median; box limits indicate the 25% and 75% quantiles; whiskers represent the 1.5× interquartile range; and points above or below whiskers represent outliers

Relative cytotoxic lymphocyte subpopulation abundance was used as the cellular TSH biomarker to separate patients into good- and poor-survival groups (threshold ratio, 0.3512). There was no significant difference in the cytotoxic lymphocyte subpopulation between the tumor and stromal tissues in the good-survival group (mean 0.9233 ± 0.36056 vs. 0.8628 ± 0.2787, Wilcoxon signed rank p = 0.455), whereas in the poor- survival group, this cell subtype was significantly higher in the stroma than in the tumors (0.9631 ± 0.2135 vs. 0.3531 ± 0.1437, Wilcoxon signed rank p = 7.755 × 10^–16^) (Fig. 4a). This cell subpopulation was positively associated with good survival in breast cancer patients (*p* < 0.0001) (Fig. 4b). This means that the tumor tissues in the good-survival group had higher levels of various cell subpopulations. Additionally, all cell subpopulation abundances were significantly lower in the tumor than in the stroma in the poor-survival group (Fig. 4c), whereas the opposite trend was observed in the good-survival group (Fig. 4d). A stronger correlation of cytotoxic lymphocyte cell subpopulation abundance between the tumor and stroma (*r* = 0.45, *p* = 0.003) was observed in the good-survival group, whereas a weaker correlation (*r* = 0.11, *p* = 0.92) was found in the poor-survival group (Additional file 1: Figure S1). The findings that the cytotoxic lymphocyte cell subpopulation is associated with survival were consistent with previous studies [46–48].

### Analysis of tumor-stroma heterogeneity by cell subpopulation networks

**Fig. 5:**
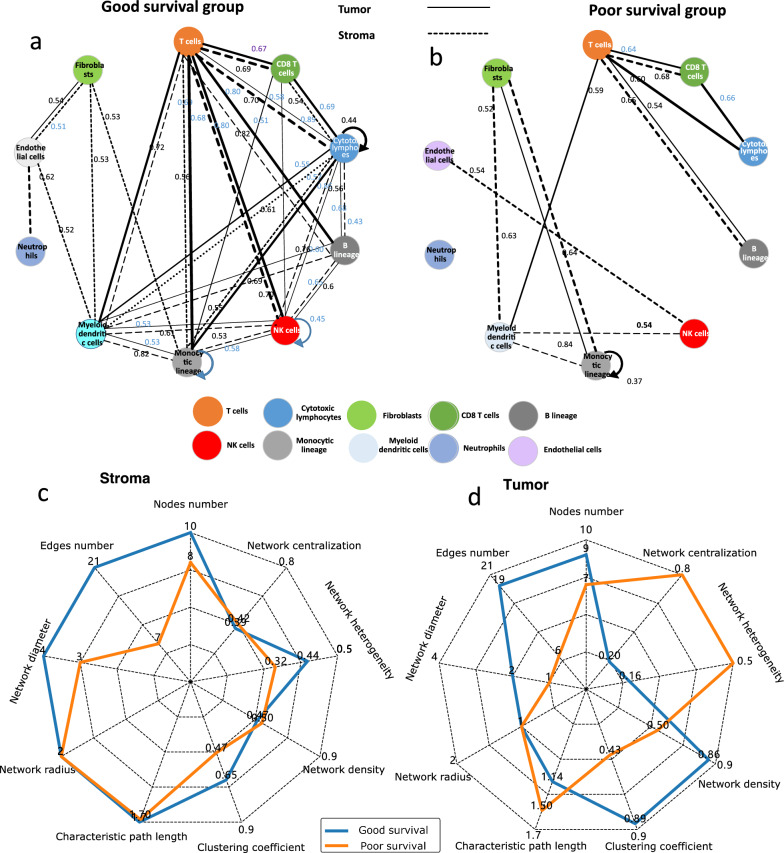
Network topology in the poor-survival and good-survival groups. **a** The cell subpopulation network in the good-survival group; **b** The cell subpopulation network in the poor-survival group; **c** The radar map of the topological parameters (n = 9) of the stroma-based cell subpopulation network for good- and poor-survival groups; **d** The radar map of the topological parameters (n = 9) of the tumor-based cell subpopulation network for good- and poor-survival groups

Both the tumor and stroma exhibited distinct cell subpopulation networks in the good-survival and poor-survival groups, as depicted in Fig. 5a and b. Notably, the stroma-based cell subpopulation network displayed dense intrinsic connectivity, while the tumor-based network exhibited sparse connectivity. Furthermore, the topological parameters (n = 9) of the cell subpopulation network differed significantly between the poor and good-survival groups for both stromal and tumor-based networks, as illustrated in Fig. 5c, d, and Additional file 1: Table S5.

In the stroma-based network, the good-survival group demonstrated a higher network diameter and greater node and edge numbers than the poor-survival group. Conversely, within the tumor-based network, the good-survival group exhibited a higher network edge number, density, and clustering coefficient than the poor-survival group. Interestingly, the good-survival group displayed relatively lower network centralization, indicating a greater dispersion of node centrality scores throughout the network in comparison to the maximum centrality score obtained. This finding suggests enhanced communication among cells, particularly T cells, to the central node in patients with poor survival.

### Imaging cellular tumor-stroma heterogeneity

**Fig. 6:**
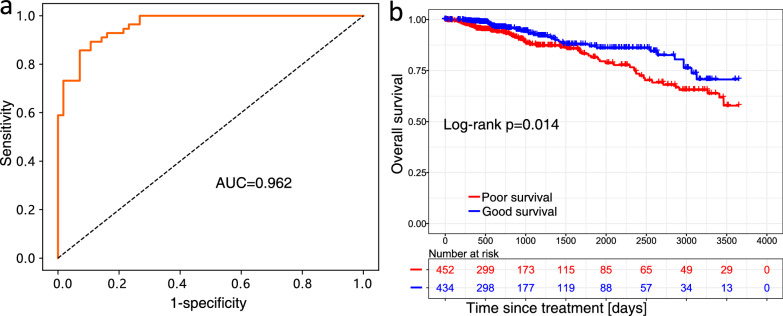
Radiogenomic analysis and predictive model based on differences between the tumor and stroma. **a** The ROC curve for the predictive model. **b** The survival curves for the tumor-stromal validation dataset to evaluate the effectiveness of the identified relative cell subtype features

To establish a predictive model for the TSH score, we employed cell subpopulations derived from tumors. Figure 2 depicts a schematic representation of the radiogenomic analysis conducted on cellular TSH. Utilizing cross-validation-based feature selection, cytotoxic lymphocyte and fibroblast subpopulations were identified as key predictors targeting TSH, yielding an AUC of 0.962, with a sensitivity and specificity of 0.893 (Fig. 6a). To ascertain the reliability of our correlation map, we proceeded to validate the predictive model using an independent tumor-stroma dataset. Remarkably, the TSH score predicted by this model significantly stratified patients into distinct good and poor-survival groups (p = 0.014) (Fig. 6b).

Subsequently, we applied the TSH model to the radiogenomic development dataset utilizing estimated cell subpopulation data derived from tumors to predict the TSH score. Notably, the predicted TSH score successfully separated patients within the radiogenomic development dataset into distinct good-survival (n = 46) and poor-survival (n = 45) groups, using a threshold of 0.5. To establish an imaging feature-based predictive model for the classification of these groups based on the TSH score, we employed an optimal subset of imaging features, constituting the radiogenomic signature of TSH.

To identify this optimal subset of imaging features, we employed a genetic algorithm-based feature selection method with a population size of 100 and 100 iterations under tenfold cross-validation. Following feature selection, the predictive model retained six features, as outlined in Additional file 1: Table S6. Among these features, three were extracted from tumors, encompassing two morphological features (flatness and sphericity) and two texture features (long-run high gray level emphasis extracted from the precontrast image and inverse difference moment normalized (IDMN) extracted from the postcontrast image). Additionally, two features were extracted from the stroma, including the IDMN feature and gray level nonuniformity (GLSZM).

The radiogenomic signatures identified in our study serve as surrogate markers for cellular TSH and hold significant prognostic value. These signatures provide informative assessments for patient prognosis, reflecting the potential of radiogenomics in enhancing our understanding of tumor-stroma interactions and facilitating personalized treatment decisions.

### Prognostic validation of the radiogenomic signatures

**Fig. 7:**
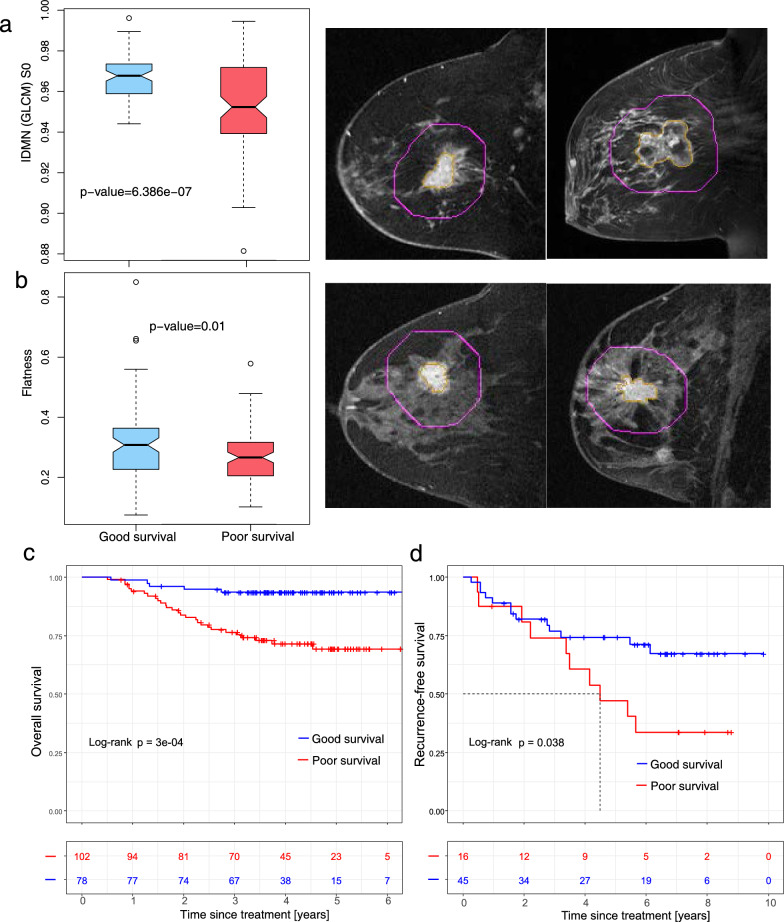
Imaging feature distribution and Kaplan–Meier survival analyses. **a** Boxplot of the inverse difference moment normalized (IDMN) features in precontrast images showing significantly higher values in the good survival group than in the poor-survival group (p = 6.386 × 10^–7^). An example of a patient aged 54.16 years showed a higher feature value (0.9657) with good survival compared with a patient aged 44.5 years with poor survival (feature value = 0.9494). **b** Boxplot of the flatness feature showed significantly higher values in the good-survival group than in the poor-survival group (p = 0.01). Examples of a patient aged 62.4 years showed a higher feature value of 0.3086 with good survival than a patient aged 58.89 years with a feature value of 0.2700 with poor survival. The predicted tumor-stroma heterogeneity (TSH) score using radiogenomic signatures (n = 6) separated patients into good- and poor-survival groups for **c** overall survival (prognostic validation 1 dataset) and **d** recurrence-free survival (prognostic validation 2 dataset)

The identified radiogenomic signatures underwent independent validation through survival analysis using two prognostic validation datasets: validation dataset 1 (n = 180) and validation dataset 2 (n = 61). Representative examples of the IDMN and flatness features are illustrated in Fig. 7a and b, respectively. The IDMN feature exhibited a significantly higher value (p = 6.386 × 10^–7^) in the good-survival group than in the poor-survival group (prognostic validation 1 dataset, Fig. 7a). Moreover, elevated IDMN values or enhanced tumor flatness were indicative of a favorable prognosis in the prognostic validation 2 dataset (Additional file 1: Figure S2).

The radiogenomic signatures identified in this study were used to generate a predicted TSH score. Analysis of this score revealed a significant positive correlation with both overall survival (OS) (p = 0.038) and recurrence-free survival (RFS) (p = 3 × 10^–4^) in the prognostic validation 1 and prognostic validation 2 datasets, as illustrated in Fig. 7c and d, respectively. After adjusting for confounding factors such as age, HER2 status, ER status, PR status, and longest diameter, the radiogenomic signature maintained its significant prognostic value for OS (p = 0.004).

The validation of the identified radiogenomic signatures in two independent prognostic validation datasets strengthens their prognostic value. Therefore, the significant association between the predicted TSH score and both OS and RFS, coupled with its independent prognostic value, highlights the relevance and reliability of the radiogenomic signatures identified in our study.

## Discussion

In this multicohort study, we identified the radiogenomic signatures of TSH and independently evaluated their clinical implications. The cellular TSH biomarker was determined by assessing the relative differences in its abundance within the tumor and stromal cell subpopulations. These cellular TSH biomarkers demonstrated their effectiveness in differentiating patients into distinct groups with varying survival rates. To further validate the clinical significance of our findings, we developed a radiogenomic signature-based model targeting TSH and tested its predictive capabilities in two independent datasets. Overall, our study demonstrates the potential of radiogenomic analysis in uncovering hidden tumor phenotypes and highlights the promising role of these imaging-based surrogates in enhancing the accuracy of prognosis and treatment management.

We observed a positive correlation between cell subpopulation abundances estimated from the surrounding nontumoral tissue and poor survival, which is in line with previous findings [45]. In the network analysis of cell subpopulations, the good-survival group exhibited a denser interconnection among themselves compared to the poor-survival group, both in the tumor- and stroma-based cell subpopulation networks. A possible explanation is that an imbalanced microenvironment can influence tumor growth and progression in breast cancer [49–52]. The observed positive correlation between cell subpopulation abundances from the surrounding nontumoral tissue and poor OS, as well as the differential interconnectivity patterns within cell subpopulation networks, provides valuable insights into the complex microenvironment of breast cancer. Further investigations into the underlying mechanisms governing these observed network characteristics hold promise for identifying novel therapeutic strategies aimed at improving patient outcomes.

In our study, we observed a stronger correlation of cytotoxic lymphocyte cell subpopulation abundance between the tumor and stroma in the good-survival group, while a weaker correlation was found in the poor-survival group. Cytotoxic lymphocytes are essential in tumor immunity due to their correlation with tumor size, overall survival, immune checkpoint expression, and tumor microenvironment characteristics [46, 48]. The higher abundance of cytotoxic lymphocyte cell subpopulations in the tumor compared to the stroma can be attributed to enhanced tumor immune surveillance, increased tumor cell killing, activation of immune checkpoints, induction of antitumor immune memory, and modulation of the tumor microenvironment, all of which foster antitumor immune responses and inhibit tumor growth [53].

To comprehensively assess tumor-stroma phenotypes, we conducted an evaluation of radiogenomic signatures derived from both the tumor and surrounding parenchyma tissues. Specifically, higher tumor flatness and sphericity values are associated with poor survival, which is consistent with previous findings [54]. These features are related to a more irregular tumor shape or a higher level of image heterogeneity. Furthermore, we observed a significantly higher IDMN value in the stroma of the good-survival group than in the poor survival group. Our radiogenomic model employs radiogenomic signatures to estimate cellular TSH, which offers a noninvasive approach to evaluate the entire tumor and its surrounding stroma. This imaging-based evaluation can be repeated throughout the course of treatment, providing valuable longitudinal information. Importantly, our study demonstrates the feasibility of utilizing imaging signatures of TSH with biological significance, eliminating the need for genomic data from tumors or stromal tissues. By leveraging noninvasive imaging, we have the opportunity to provide valuable insights into the tumor-stroma interplay and its impact on patient outcomes, without the need for invasive biopsies at multiple sites.

Previous studies have investigated the association between radiomic features and the immune microenvironment in terms of TILs [26, 55]. Sun et al. developed a radiomic signature of CD8 T cells, which demonstrated an association with clinical response and outcomes in a cohort of 137 patients treated with anti-PD1 immunotherapy. Moreover, this signature was subsequently employed to predict lesion response in 94 patients who underwent combined immunotherapy and radiotherapy [28]. A related study developed and validated a stromal imaging signature that could accurately diagnose and predict the survival benefit of adjuvant chemotherapy in resected gastric cancer [29]. Notably, our study differs from previous investigations, as we integrated imaging and matched cell subpopulation data from both within and outside the tumor and applied them to external datasets for survival analysis.

This study has some limitations. First, this was a retrospective study and may have been subject to patient selection bias. Second, the imaging data were collected from multiple cohorts, each with different imaging parameters. Therefore, it is crucial to conduct rigorous testing in future studies to assess the generalizability of the proposed imaging signatures in clinical applications. Third, the estimation of cell subpopulation abundance in this study relied on a deconvolution method utilizing mRNA data. It is worth noting that the accuracy of this method may impact the identification of cellular biomarkers and should be taken into consideration. Third, the data size is limited in this study. It is also important to note that acquiring both gene expression and imaging data simultaneously is particularly challenging and the availability of such data is limited. We have focused on obtaining paired gene expression and imaging data to train our models. This approach allows us to establish a correlation between the molecular characteristics represented by gene expression and the corresponding visual features observed through imaging techniques.

To address the critical challenge of incorporating prognostic information embedded in tumor-stroma heterogeneity, our predictive model utilizes novel radiogenomic signatures. These signatures capture the latent associations between the prognostic genomic signatures of TSH and radiomic features. As a result, these signatures have the potential to significantly enhance the accuracy of clinical prognostication with biological meanings. By providing valuable insights, our approach holds promise for improving patient management and guiding treatment decisions.

## Conclusions

We investigated cellular TSH and identified prognostic factors that reflect the environmental imbalance between the tumor and stroma. The relative abundance of cytotoxic lymphocyte subpopulations was used as a biomarker for cellular TSH, allowing for the stratification of patients into distinct survival groups. The good-survival group exhibited a more densely interconnected network of cell subpopulations, either within the tumor or stroma, than the poor-survival group. These cell biomarkers, identified through radiological imaging signatures, have breast cancer-related biological implications and demonstrate prognostic power. The radiogenomic signature of cellular TSH was positively associated with good survival. External validation using multicohort datasets further supports their clinical relevance for prognostic assessments. This proposed model has the potential to refine prognosis analysis and guide personalized therapy for breast cancer patients who are likely to benefit from chemotherapy and have a favorable prognosis. Our framework could be extended to other cancer types to identify patients at high risk of recurrence. Further studies are required to confirm the clinical implications and establish the clinical utility of this approach.

### Supplementary Information

**Additional file 1: Figure S1.** Relative values of the cytotoxic lymphocyte cell subpopulations. **Figure S2.** Distributions of imaging features in the good survival and poor survival groups. Significantly higher a) inverse difference moment normalized (IDMN) feature values in precontrast images and b) significantly higher tumor flatness values in the good survival group than in the poor survival group. **Table S1.** Imaging feature list. **Table S2.** Tumor cell subpopulations associated with survival in breast cancer. **Table S3.** Stromal cell subpopulations associated with survival in breast cancer. **Table S4.** Multivariate analysis of tumor/stroma subpopulations associated with survival in breast cancer. **Table S5.** Network characteristics of cell-to-cell connections. **Table S6.** Radiogenomic signatures in the predictive model.

## Data Availability

The gene expression data of tumor-stroma development and tumor-stroma validation datasets are available from the TCGA-BRCA project of Genomic Data Commons. [https://portal.gdc.cancer.gov/projects/TCGA-BRCA]. The imaging data of the tumor-stroma development and tumor-stroma validation datasets are available from TCGA-BRCA in TCIA on the website. [https://wiki.cancerimagingarchive.net/display/Public/TCGA-BRCA]. Breast-MRI-NACT-Pilot is available in TCIA on the website. [https://wiki.cancerimagingarchive.net/display/Public/Breast-MRI-NACT-Pilot]. The ISPY-1 trial is available in TCIA on the website. [https://wiki.cancerimagingarchive.net/display/Public/ISPY1].

## Abbreviations

TSH: Tumor-stroma heterogeneity
AUC: Area under the ROC curve
TILs: Tumor-infiltrating lymphocytes
DCE-MRI: Dynamic contrast-enhanced magnetic resonance imaging
HR: Hazard ratio
OS: Overall survival
RFS: Recurrence-free survival
