# Supplementary material for: Radiogenomic analysis of cellular tumor-stroma heterogeneity as a prognostic predictor in breast cancer
Source: J Transl Med. 2023 Nov 25;21:851. doi: 10.1186/s12967-023-04748-6 (PMC10675940; doi:10.1186/s12967-023-04748-6)
Supplement: Supplementary file 1 — Additional file 1: Figure S1. Relative values of the cytotoxic lymphocyte cell subpopulations. Figure S2. Distributions of imaging features in the good survival and poor survival groups. Significantly higher a) inverse difference moment normalized (IDMN) feature values in precontrast images and b) significantly higher tumor flatness values in the good survival group than in the poor survival group. Table S1. Imaging feature list. Table S2. Tumor cell subpopulations associated with survival in breast cancer. Table S3. Stromal cell subpopulations associated with survival in breast cancer. Table S4. Multivariate analysis of tumor/stroma subpopulations associated with survival in breast cancer. Table S5. Network characteristics of cell-to-cell connections. Table S6. Radiogenomic signatures in the predictive model. [file 12967_2023_4748_MOESM1_ESM.docx]

**Additional information**

**Figures**

**Figure S1.** Relative values of the cytotoxic lymphocyte cell subpopulations.

**Figure S2**. Distributions of imaging features in the good survival and poor survival groups. Significantly higher a) inverse difference moment normalized (IDMN) feature values in precontrast images and b) significantly higher tumor flatness values in the good survival group than in the poor survival group.

**Tables**

**Table S1.** Imaging feature list

| Feature category | Feature name |
| --- | --- |
| Statistical feature (n=18) | Energy, entropy, minimum, 10 percentile, 90 percentile, maximum, mean, median, interquartile range, range, mean absolute deviation, root mean squared, total energy, robust mean absolute deviation, skewness, kurtosis, variance, uniformity |
| Morphologic feature (n=14) | Elongation, flatness, least axis length, major axis length, maximum 2D diameter column, maximum 2D diameter row, maximum 2D diameter slice, maximum 3D diameter, mesh volume, minor axis length, sphericity, surface area, surface volume ratio, voxel volume |
| Gray level cooccurrence matrix-based features (n=24) | Autocorrelation, contrast, correlation, difference average, difference entropy, difference variance, joint energy, joint entropy, informational measure of correlation 1, informational measure of correlation 2, inverse difference moment, maximal correlation coefficient, inverse difference moment normalized, inverse difference, inverse difference normalized, inverse variance, maximum probability, sum average, sum entropy, sum squares |
| Gray level size zone matrix-based features (n=16) | Small area emphasis, large area emphasis, gray level nonuniformity, gray level nonuniformity normalized, size zone nonuniformity, size zone nonuniformity normalized, zone percentage, gray level variance, zone variance, zone entropy, low gray level zone emphasis, high gray level zone emphasis, small area low gray level emphasis, small area high gray level emphasis, large area low gray level emphasis, large area high gray level emphasis |
| Gray-Level run length matrix-based features (n=16) | Short run emphasis, long run emphasis, gray level nonuniformity, gray level nonuniformity normalized, run length nonuniformity, run length nonuniformity normalized, run percentage, gray level variance, run variance, run entropy, low gray level run emphasis, high gray level run emphasis, short run low gray level emphasis, short run high gray level emphasis, long run low gray level emphasis, long run high gray level emphasis |
| Gray level difference matrix-based features (n=14) | Small dependence emphasis, large dependence emphasis, gray level nonuniformity, dependence nonuniformity, dependence nonuniformity normalized, gray level variance, dependence variance, dependence entropy, low gray level emphasis, high gray level emphasis, small dependence low gray level emphasis, small dependence high gray level emphasis, large dependence low gray level emphasis, large dependence high gray level emphasis |
| Neighborhood gray tone difference matrix (n=5) | Coarseness, contrast, busyness, complexity, strength |

**Table S2**. Tumor cell subpopulations associated with survival in breast cancer

| Feature | Beta | HR (95%CI) | Wald test | p value | Corrected p |
| --- | --- | --- | --- | --- | --- |
| **T cells** | **-1.30** | **0.27 (0.132-0.558)** | **12.6** | **0.0004** | **0.0016** |
| **CD8 T cells** | **-0.53** | **0.59 (0.394-0.877)** | **6.77** | **0.0093** | **0.017** |
| **Cytotoxic lymphocytes** | **-1.81** | **0.16 (0.060-0.449)** | **12.38** | **0.0004** | **0.0016** |
| **B lineage** | **-1.05** | **0.35 (0.176-0.697)** | **8.94** | **0.0028** | **0.0061** |
| NK cells | -3.43 | 0.03 (0.001-0.919) | 4.04 | 0.0445 | 0.07 |
| Monocytic lineage | -0.38 | 0.68 (0.390-1.194) | 1.8 | 0.1803 | 0.25 |
| **Myeloid dendritic cells** | **-0.94** | **0.39 (0.217-0.700)** | **9.92** | **0.0016** | **0.0045** |
| Neutrophils | -0.76 | 0.47 (0.088-2.485) | 0.8 | 0.3723 | 0.455 |
| Endothelial cells | -0.01 | 0.99 (0.505-1.938) | 0 | 0.9738 | 0.9738 |
| Fibroblasts | -0.14 | 0.87 (0.623-1.215) | 0.67 | 0.4137 | 0.455 |

**Table S3.** Stromal cell subpopulations associated with survival in breast cancer

| Feature | Beta | HR (95%CI) | Wald | p value | Corrected p |
| --- | --- | --- | --- | --- | --- |
| T cells | -0.75 | 0.47 (0.152-1.477) | 1.66 | 0.198 | 0.476 |
| CD8 T cells | -0.33 | 0.72 (0.415-1.235) | 1.44 | 0.230 | 0.476 |
| Cytotoxic lymphocytes | 0.35 | 1.42 (0.435-4.636) | 0.34 | 0.562 | 0.687 |
| B lineage | -0.58 | 0.56 (0.207-1.529) | 1.27 | 0.259 | 0.476 |
| NK cells | 1.83 | 6.25 (0.111-350.887) | 0.8 | 0.372 | 0.512 |
| Monocytic lineage | 0.12 | 1.12 (0.618-2.046) | 0.15 | 0.700 | 0.770 |
| Myeloid dendritic cells | -0.13 | 0.87 (0.355-2.155) | 0.09 | 0.770 | 0.770 |
| **Neutrophils** | **2.31** | **10.07 (1.964-51.587)** | **7.67** | **0.006** | **0.031** |
| Endothelial cells | 0.60 | 1.82 (0.879-3.775) | 2.6 | 0.107 | 0.391 |
| Fibroblasts | 0.25 | 1.28 (0.790-2.077) | 1.01 | 0.315 | 0.495 |

**Table S4**. Multivariate analysis of tumor/stroma subpopulations associated with survival in breast cancer

| Feature | Beta | HR (95%CI) | Wald test | p value |
| --- | --- | --- | --- | --- |
| T cells | -0.34 | 0.71 (0.301-1.699) | -0.76 | 0.447 |
| **Cytotoxic lymphocytes** | -2.01 | 0.13 (0.026-0.700) | -2.38 | 0.017 |
| B lineage | 0.12 | 1.13 (0.618-2.062) | 0.39 | 0.695 |
| NK cells | 0.57 | 1.76 (0.794-3.899) | 1.39 | 0.163 |
| Myeloid dendritic cells | -0.15 | 0.86 (0.368-2.002) | -0.35 | 0.723 |

**Table S5.** Network characteristics of cell-to-cell connections

| Topological parameters | Normal | Tumor | Normal | | Tumor | |
| --- | --- | --- | --- | --- | --- | --- |
|  |  |  | Good survival | Poor survival | Good survival | Poor survival |
| Nodes number | 10 | 9 | 10 | 8 | 9 | 7 |
| Edges number | 12 | 20 | 21 | 7 | 19 | 6 |
| Network diameter | 3 | 2 | 4 | 3 | 2 | 2 |
| Network radius | 2 | 1 | 2 | 2 | 1 | 1 |
| Characteristic path length | 1.714 | 1.095 | 1.733 | 1.7 | 1.143 | 1.5 |
| Clustering coefficient | 0.658 | 0.924 | 0.646 | 0.467 | 0.886 | 0.433 |
| Network density | 0.393 | 0.905 | 0.467 | 0.5 | 0.857 | 0.5 |
| Network heterogeneity | 0.538 | 0.134 | 0.436 | 0.316 | 0.162 | 0.548 |
| Network centralization | 0.619 | 0.133 | 0.389 | 0.417 | 0.2 | 0.833 |

**Table S6.** Radiogenomic signatures in the predictive model

| Sequence | Position | Feature name | Feature category |
| --- | --- | --- | --- |
| S_0_ | Tumor | Flatness | Shape |
| S_0_ | Tumor | Sphericity | Shape |
| S_0_ | Tumor | Long Run High Gray Level Emphasis | Texture |
| S_0_ | Stroma | Inverse difference moment normalized | Texture |
| S_0_ |  | Gray Level Nonuniformity | Texture |
| S_I_ | Tumor | Inverse difference moment normalized | Texture |

S_0_ and S_I_ denote the precontrast and the subtraction between the intermediate image series and S_0_.
